# A multicenter, randomized controlled trial to compare the effectiveness of STARC-SUD (Skills Training in Affect Regulation – a Culture-sensitive approach) versus treatment as usual in trauma-exposed refugees with substance use problems

**DOI:** 10.1186/s13063-022-06761-4

**Published:** 2022-10-28

**Authors:** Ingo Schäfer, Philipp Hiller, Sascha Milin, Annett Lotzin

**Affiliations:** 1 Department of Psychiatry and Psychotherapy, University Medical Center Hamburg Eppendorf, Hamburg, Germany; 2 University Medical Center Hamburg-Eppendorf, Hamburg, Germany

**Keywords:** Substance use disorders, Substance abuse, Trauma exposure, Refugees, Randomized controlled trial, Clinical trial, Transdiagnostic behavior therapy, Emotion regulation, Group intervention, Protocol

## Abstract

**Background:**

Refugees often report high levels of psychological distress due to traumatic experiences before and during flight as well as many post-migration stressors. Refugees with hazardous substance use or existing substance use disorder (SUD) are a particularly vulnerable group for whom few preventive and therapeutic measures are available. The aim of this study is to investigate the effectiveness of an integrative culturally sensitive group therapy approach (STARC-SUD) to improve affect regulation in refugees with substance-related problems.

**Methods:**

The study aims to include *N* = 286 male refugees with psychological distress (GHQ-12 > 13) and hazardous substance use or SUD (AUDIT > 7 or DUDIT > 6). Therapists working supported by interpreters will deliver the STARC-SUD intervention in addiction aid facilities in six metropolitan regions of Germany. The primary endpoint is severity of psychological distress (GHQ-12). The effectiveness of STARC-SUD is compared with treatment as usual (TAU) post-intervention and 3 months later.

**Discussion:**

This trial will be one of the first RCTs on a culturally sensitive transdiagnostic intervention for trauma-exposed refugees with hazardous substances or SUD. The trial might gain new insights into the efficacy of such an intervention.

**Trial registration:**

OSF Registry osf.io/nhxd4. Registered prospectively on September 22, 2020, doi: 10.17605/OSF.IO/NHXD4. DRKS DRKS00017668

## Administrative information

Note: The numbers in curly brackets in this protocol refer to the SPIRIT checklist item numbers. The order of the items has been modified to group similar items (see http://www.equator-network.org/reporting-guidelines/spirit-2013-statement-defining-standard-protocol-items-for-clinical-trials/).

**Table:** 

Title {1}	A multicenter, randomized controlled trial to compare the effectiveness of STARC (“Skills Training in Affect Regulation - a culture-sensitive approach”) versus Treatment as Usual in trauma-exposed refugees with substance use problems
Trial registration {2a and 2b}.	Registered prospectively on September 22, 2020. OSF registry osf.io/nhxd4, doi: 10.17605/OSF.IO/NHXD4.
Protocol version {3}	Issue date: 8 NOV 2021 Protocol amendment number: Not applicable Authors: AL
Funding {4}	The Federal Ministry of Education and Research (BMBF) (01EF1805A).
Author details {5a}	Ingo Schäfer, Department of Psychiatry and Psychotherapy, University Medical Center Hamburg Eppendorf, Hamburg, Germany Philipp Hiller, University Medical Center Hamburg-Eppendorf, Germany Sascha Milin, Department of Psychiatry and Psychotherapy, University Medical Center Hamburg Eppendorf, Hamburg, Germany Annett Lotzin, Department of Psychiatry and Psychotherapy, University Medical Center Hamburg Eppendorf, Hamburg, Germany
Name and contact information for the trial sponsor {5b}	Trial Sponsor: University Medical Center Hamburg-Eppendorf Sponsor’s Reference: 01EF1805A. Contact name: Prof. Dr. med. Ingo Schäfer, MPH Address: Department of Psychiatry and Psychotherapy, University Medical Center Hamburg-Eppendorf, Martinistr. 52 20246 Hamburg Telephone: +49-(0)40-7410-59290; Email: i.schaefer@uke.de
Role of sponsor {5c}	The sponsor—University Medical Center Hamburg-Eppendorf—is responsible for the design of the study; the collection, analysis, and interpretation of the data; and the writing of the manuscript.

## Introduction

### Background and rationale {6a}

Relationships between hazardous substance use or substance use disorders (SUD) and mental disorders have been well documented [1]. Individuals with comorbid mental disorders related to post-traumatic stress may find themselves rejected from both mental health care programs and addiction services [2]. It has therefore been recommended to integrate SUD services for refugees with mental health services when offering care for refugees with SUD [3]. Adequate interventions for refugees with hazardous substance use or SUD and co-occurring post-traumatic psychological distress are urgently needed.

Very few studies investigated the effects of substance use interventions in refugee populations. A recent randomized controlled trial (RCT) among Somali refugees living in Kenya suggested small effects of a standardized brief intervention to reduce Khat use in this population [4]. The intervention was less effective in participants with comorbid psychopathology, particularly in patients with PTSD. Another RCT examined the effects of the “Common Elements Treatment Approach (CETA),” a transdiagnostic treatment for comorbid disorders in refugees in Thailand [5]. Moderate to large symptom reductions were observed for depression, anxiety, and post-traumatic stress, but not for alcohol use. Taken together, the existing studies suggest that more evidence on the efficacy of interventions for refugees with SUD is needed. Neither a brief SUD treatment alone nor a psychotherapeutic approach without SUD treatment had a sufficient effect on both, problematic substance use and comorbid psychological symptoms.

In non-migrant populations with SUD and post-traumatic symptoms, low-threshold integrative interventions are effective in reducing psychological distress and emotion dysregulation. For example, a recent RCT on the effects of a low-threshold stabilizing integrated group treatment for SUD and PTSD in a non-migrant population was compared to usual SUD relapse prevention and to a waitlist control group for women with SUD and PTSD [6]. The results of this study revealed a significantly greater reduction in psychological distress in patients that received the integrative intervention compared to patients on the waitlist at post-treatment (*d* = 0.39), which was sustained at 3- and 6-month follow-up. Such interventions should be culturally adapted and, given the spectrum of psychological symptoms among refugees (e.g., PTSD, anxiety disorders, depression [7]), take a transdiagnostic approach that targets underlying vulnerabilities (e.g., [8]).

Recently, a culture-sensitive transdiagnostic cognitive-behavioral group therapy for refugees (STARC) has been developed that aims at reducing psychological distress by focussing on emotion regulation and has been successfully tested for feasibility [9]. However, refugees with hazardous substance use or SUD might need an intervention that additionally considers problematic substance use or SUD. However, such an intervention has not been developed and evaluated yet. Therefore, the aim of this study is to develop and examine the effects of a transdiagnostic integrative group intervention for refugees with hazardous substance use or SUD and co-occurring psychological distress (STARC-SUD) on reducing refugees’ psychological distress.

### The need for a trial

Both addiction treatment facilities and institutions providing mental health care for refugees in Germany are confronted with the challenge to provide adequate care for individuals with psychological distress as a consequence of traumatic experiences and additional hazardous substance use or SUD that complicates treatment. Adequate interventions for this target group are urgently needed. If the “Skills Training in Affect Regulation – a Culture-sensitive Approach in refugees with Substance Use Disorder” (STARC) intervention, adapted for refugees with substance use or SUD, will prove to be effective, the intervention might help to provide evidence-based treatment for refugees with trauma exposure and substance use problems.

### Objectives {7}

The primary objective is to determine if STARC-SUD is more effective in reducing psychological distress in refugees with trauma exposure and substance use problems. It is hypothesized that STARC-SUD is more effective in reducing psychological distress than treatment as usual, i.e., the care that they would usually receive from SUD and/or refugee counseling or treatment centers. The secondary objective is to compare the effectiveness of STARC-SUD versus treatment as usual in reducing emotion dysregulation, substance use, and symptoms of PTSD.

### Trial design {8}

This study is a multicenter, randomized controlled, outcome adjudicator-blinded superiority trial with two parallel groups and a primary endpoint of psychological distress post-treatment to compare the effectiveness of STARC SUD (“Skills Training in Affect Regulation – a culture-sensitive approach”) versus treatment as usual in trauma-exposed refugees with substance use problems. The randomization will be performed in blocks of 8 with a 1:1 allocation to one of the two treatment arms. Randomization numbers will be assigned to eligible study participants in ascending order of their inclusion.

## Methods: participants, interventions, and outcomes

### Study setting {9}

Participants will be recruited in six metropolitan regions of Germany (Hamburg, Bremen, Berlin, Hannover, Frankfurt, München). The intervention is conducted in SUD counseling and treatment centers in the six metropolitan regions.

### Eligibility criteria {10}

The following are the inclusion criteria: (1) psychological distress (GHQ-12 > 13); (2) hazardous substance or SUD (AUDIT > 7, DUDIT > 6); (3) exposure to traumatic experiences (e.g., war, persecution, torture, or traumatic experiences during flight; PCL-5); (4) asylum seeker or refugee (e.g., Afghanistan, Syria, Iraq); and (6) male gender. The following are the exclusion criteria: (1) acute psychosis and (2) acute suicidality.

### Who will take informed consent? {26a}

Information sheets about the study and consent forms are provided for all participants involved in the trial. The information sheets and consent forms have been written in easy language and have been translated into Arabic, Farsi, and English. The research personnel will introduce the trial and inform the participants about its main aspects. The research personnel will then discuss the trial with the participants in light of the information provided. If the participant is eligible and willing to participate in the trial, the research personnel will obtain written consent from the participant.

### Additional consent provisions for collection and use of participant data and biological specimens {26b}

On the consent form, participants will be asked if they agree to the use of their data. Participants will be asked for permission for the research team to share relevant data with people taking part in the research or from regulatory authorities, where relevant. This trial does not involve collecting biological specimens.

### Interventions

#### Explanation for the choice of comparators {6b}

A treatment as usual (TAU) control group is used to compare the experimental intervention to treatments that are already used in clinical practice. Patients in the TAU group will not be offered any additional specific intervention other than the care that they would usually receive from SUD and/or refugee counseling or treatment centers or their general practitioner.

#### Intervention description {11a}

The intervention (STARC-SUD) is tailored to the needs of refugees with SUD and additional psychological distress. STARC-SUD is an adaption of Skills-Training of Affect Regulation – a Culture-sensitive Approach (STARC) [10], a culturally sensitive group intervention developed for refugees in Western middle- or high-income countries. The intervention is based on elements from skills-based treatments (e.g., Skills Training in Affective and Interpersonal Regulation therapy (STAIR [11];), Dialectic Behavioral Therapy (DBT [12];), and Culturally Adapted Cognitive Behavioral Therapy (CA-CBT [13];)). The authors developed the STARC program according to guidelines for developing culturally sensitive interventions [14]. The manual includes culture-sensitive metaphors and expressions and uses easy language. A pilot study on Afghan refugees indicated preliminary evidence that the intervention reduces difficulties in emotion regulation, general distress, and post-traumatic stress disorder symptoms [9].

For this trial, STARC was adapted for refugees with SUD [15]. The conceptual framework of Heim and Kohrt was used for the adaptions [16]. The results of five focus group discussions with refugees on concepts of SUD and their treatment informed the adaption. An expert group suggested adaptions and decided by consensus on their implementation. Two pilot groups were conducted with the adapted STARC-SUD program. Interviews with the therapists of these pilot groups informed further adaption.

#### Criteria for discontinuing or modifying allocated interventions {11b}

In case of a serious adverse event, the PI will decide together with a Study Safety Board if the treatment needs to be discontinued. Participants are free to discontinue or modify the allocated intervention at any time of the study. In case of worsening disease (e.g., acute suicidality), the study personnel will consult the participant on additional necessary treatment and referral options.

#### Strategies to improve adherence to interventions {11c}

All study therapists were trained in the provision of the intervention prior to the start of the study. To ensure fidelity of treatment, the provision of treatment will be highly standardized (use of a manual, intensive training of all therapists by the authors of the STARC-SUD program, and on-going supervision). Adherence will be further assured through regular therapist meetings.

#### Relevant concomitant care permitted or prohibited during the trial {11d}

Concomitant care is permitted for both groups.

#### Provisions for post-trial care {30}

There is no anticipated harm to trial participation.

### Outcomes {12}

The primary outcome will be the severity of psychological distress as measured with the General Health Questionnaire-12 (GHQ-12) [17] at *t*_1_ (post-treatment) and *t*_2_ (3-month follow-up).

The following are the secondary outcomes: severity of emotion dysregulation will be assessed at *t*_0_, *t*_1_, and *t*_2_ using the Difficulties in Emotion Regulation Scale (DERS) [18]. Substance use will be assessed by using the Alcohol Use Disorders Identification Test (AUDIT) [19] and the Drug Use Disorders Identification Test (DUDIT) [20] at *t*_0_, *t*_1_, and *t*_2_. Substance use within the last 30 days will be assessed at *t*_0_, *t*_1_, and *t*_2_ using the Addiction Severity Index-Lite (ASI-Lite) [21]. Symptoms of PTSD will be assessed at *t*_0_, *t*_1_, and *t*_2_ using the PTSD Checklist for DSM-5 (PCL-5) [22].

#### Participant timeline {13}

Potential participants who self-refer or are referred by collaborating services will be invited for an eligibility assessment by a study therapist at one of the trial locations. Potential participants will receive information about the trial and an information sheet and will have the possibility to ask questions. The information sheet provides information about the aims of the study, the clinical assessment and random allocation process, treatment, and assessment schedule with details of time involved, potential benefits and risks of taking part, ethical approval, funding information, data management and confidentiality, freedom to withdraw at any time, financial reimbursement, and contact details. Afterwards, the potential study participant will be invited to a short interview with the study therapist to screen for major inclusion and exclusion criteria (e.g., substance use problems). Potential eligible participants will be invited to conduct a 1–2-h *t*_0_ assessment with the study personnel to assess all the inclusion and exclusion criteria and additional baseline characteristics. This will include assessment of sociodemographic variables (gender and asylum/residence status), general psychological distress (General Health Questionnaire-12 (GHQ-12)), alcohol use (Alcohol Use Disorders Identification Test (AUDIT)), substance use (Drug Use Disorders Identification Test (DUDIT)), trauma exposure (trauma exposure section of the PTSD Checklist for DSM-5 (PCL-5)), and a clinical evaluation of acute suicidality and psychosis using purpose-designed items. Eligible participants that agree to participate will sign the informed consent form for the trial. Those who are not eligible or do not agree to participate in the trial will be advised about alternative treatment options.

**Fig. 1:**
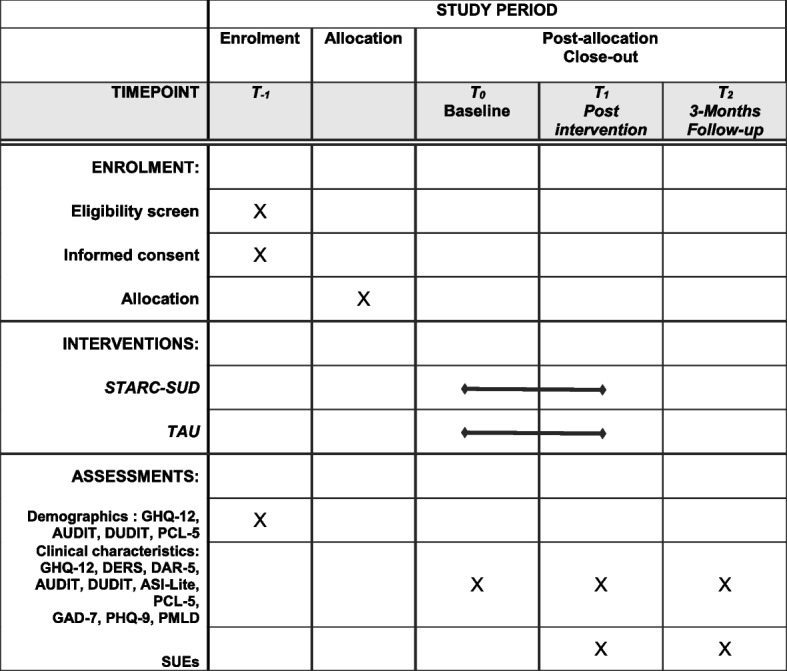
Schedule of enrollment, interventions, and assessments. GHQ-12, General Health Questionnaire-12; DERS, Difficulties in Emotion Regulation Scale, post-traumatic anger; DAR-5, Dimensions of Anger Reactions Scale-5; AUDIT, Alcohol Use Disorders Identification Test; DUDIT, Drug Use Disorders Identification Test; ASI-Lite, Addiction Severity Index-Lite; PCL-5, PTSD Checklist for DSM-5; GAD-7, Generalized Anxiety Disorder 7; PHQ-9, Patient Health Questionnaire; PMLD, Migration Living Difficulties Checklist

In addition to this *t*_0_ assessment, the participants will be assessed at end of treatment (*t*_1_) and 3 months later (*t*_2_; Fig. 1). All assessments will be conducted with e-CRFs in the language most appropriate for the respective participant (e.g., Arabic, Farsi, English, German) using tablet computers. The study personnel will assist with the data assessments. Translators will assist with the data assessment, will translate the questions if necessary, and will verify that all questions are correctly understood.

The *t*_0_, *t*_1_, and *t*_2_ assessment will include the substance use section of the Addiction Severity Index (substance used within the last 30 days), PTSD symptoms (PTSD Checklist for DSM-5 (PCL-5)), anxiety symptoms (Generalized Anxiety Disorder 7 (GAD-7)), depression symptoms (Patient Health Questionnaire (PHQ-9)), emotion dysregulation (DERS, DAR-5), quality of life (World Health Organization Quality of Life Scale (WHOQOL-BREF)), post-migration stressors (PMLD; Silove et al. (1998), and purpose-designed items on therapy expectations.

#### Sample size {14}

*N* = 286 participants are planned to be included in the study. An earlier RCT comparing a transdiagnostic group intervention for refugees vs. treatment as usual found a greater reduction in psychological distress (as measured by GHQ-12) in the intervention group compared with the control group, with a medium effect size of *d* = 0.57 in the change from baseline to 3-month follow-up [23]. A pilot study of STARC showed that the recruitment of refugees with psychological distress was feasible, with about 85% of the screened patients being eligible for the study [9].

In a previous study evaluating the effects of an integrated present-focused treatment of SUD and PTSD, we found a greater reduction in psychological distress (as measured by SCL-27) in the intervention group compared with the control group, with a mean effect size of *d* = 0.39 [6]. On the basis of these results, we assume a small to medium effect size of *d* = 0.40 for STARC-SUD compared to TAU in a sample of refugees with SUD and co-occurring psychological distress. To detect between-group differences of an effect size of *d* = 0.40 (*α* = 0.05; power = 0.80), 100 participants per group are required. To account for a dropout rate of 30%, we aim to recruit 286 participants.

#### Recruitment {15}

To include 286 individuals, participants will be recruited from SUD counseling and/or treatment centers in 6 metropolitan regions of Germany (Hamburg, Bremen, Berlin, Hannover, Frankfurt, München). Participants will be recruited by promotional print material, videos, blogs, personal presentations in refugee camps, SUD treatment, and counseling centers, via advertisements in magazines, on websites, and on social media.

Assuming that about two-thirds (67%) of the screened refugees will be eligible, and about two-thirds (67%) of the eligible refugees will agree to participate, approximately 5 refugees per month need to be screened in each center, and about 4 participants per quarter (16 per 12 months) need to be randomized at each of the 6 sites to include 286 patients. The approx. enrollment period will span 36 months.

### Assignment of interventions: allocation

#### Sequence generation {16a}

The allocation sequence was generated by computer-generated random numbers using R. Participants are randomly assigned to either the control or the experimental group with a 1:1 allocation as per a computer-generated randomization schedule stratified by site using blocks of random sizes. The block sizes will not be disclosed to ensure concealment.

#### Concealment mechanism {16b}

The random number is sequentially ordered and will be allocated in ascending order. The study personnel will allocate the next random number to the next eligible participant by accessing a web-based random platform in which the random sequence is stored. The allocation sequence is concealed, as the web-based platform will not release the randomization code until the patient has been recruited into the trial.

#### Implementation {16c}

The allocation sequence was generated by a researcher not involved in the recruitment process of this study. Participants will be enrolled after the eligibility assessment by the study personnel who will inform the participant about group allocation. A response form will be sent to the study therapist who is not involved in evaluating the results of the study. The staff responsible for the data analysis will not receive information about the group assignment.

### Assignment of interventions: blinding

#### Who will be blinded {17a}

The outcome adjudicators will be blinded for the assignment of interventions. For feasibility reasons, the data assessors will be only blinded at the *t*_0_ assessment in the participating sites. Assessors will be trained in a standardized assessment procedure (e.g., adhering to the identical questions) to reduce bias related to unblinding.

#### Procedure for unblinding if needed {17b}

Data assessors are only blinded for the *t*_0_ baseline assessment for feasibility reasons. Only the data adjudicators are blinded for all assessments and will not be unblinded before the end of the data analysis. As the remaining study personnel is unblinded, an unblinding procedure will not be necessary for this study.

### Data collection and management

#### Plans for assessment and collection of outcomes {18a}

The coordinating site will be responsible for the organization of the data assessment at the different study sites. The data management team of CIAR will prepare the study measures and perform the programming of measures for data entry via tablets, the data verification, the data cleaning, the plausibility checks, and the regular control of data quality.

Study instruments will include the General Health Questionnaire-12 (GHQ-12), Alcohol Use Disorders Identification Test (AUDIT), Drug Use Disorders Identification Test (DUDIT), PTSD Checklist for DSM-5 (PCL-5), Difficulties in Emotion Regulation Scale (DERS), Dimensions of Anger Reactions Scale-5 (DAR-5), Addiction Severity Index Lite (ASI-Lite), General Anxiety Disorder (GAD-7) Questionnaire, Patient Health Questionnaire-9 (PHQ-9), Post Migration Living Difficulties Checklist (PMLD), WHO Quality of Life Scale Abbreviated Version (WHOQOL-BREF), and serious adverse events (SAEs; German: SUEs).

Psychological distress is assessed by the General Health Questionnaire-12 (GHQ-12 [24]). It aims to measure the current and recent general health over the last few weeks. The GHQ-12 consists of 12 items which are rated on a 4-point response scale. It has shown good psychometric properties in regard to reliability and validity in general health care settings with patient samples, making this short screening instrument work as well as its longer version [24].

Alcohol use is assessed by the Alcohol Use Disorders Identification Test (AUDIT [19]). The AUDIT consists of 10 items and aims to assess past-year hazardous drinking, harmful alcohol use, and alcohol dependence. Eight items are rated on a 5-point scale, and 2 items are rated on a 3-point scale all ranging between 0 and 4. A total score can be derived from summarizing all items. Higher values represent higher levels of problematic alcohol use [19]. The AUDIT has shown good psychometric properties, such as test-retest reliability and internal consistency, in a variety of settings [25].

Drug use is assessed using the Drug Use Disorders Identification Test (DUDIT [26]). The DUDIT contains 11 items and aims to assess past-year substance use disorders of a wide variety of drugs. Nine items are rated on a 5-point scale, and 2 items are rated on a 3-point scale. The DUDIT has shown good measures of reliability and validity in clinical settings and research [27].

Symptoms of PTSD will be assessed by using the PTSD Checklist for DSM-5 (PCL-5 [22]) including the Life Events Checklist for DSM-5 (LEC-5) and Criterion A. The PCL-5 is a 20-item self-report measure assessing the 20 symptoms of PTSD described in DSM-5 over the past month. It is used for monitoring symptom change during and after treatment, screening individuals for PTSD, and making a provisional PTSD diagnosis. Items are ranked on a 5-point Likert scale ranging from 0 to 4. Sum scores will be used for analyses [22]. The PCL-5 is valid and reliable, useful in determining symptom severity, and sensitive to symptom change among military servicemembers and undergraduate student samples [22].

Emotion dysregulation will be monitored by using the Difficulties in Emotion Regulation Scale (DERS [18]). Participants rate all of the 36 items on a 5-point Likert scale. The scale focuses on adaptive ways of responding to emotional distress and can be categorized into 6 subscales ((a) lack of emotional awareness, (b) lack of emotional clarity, (c) difficulty regulating behavior when distressed, (d) difficulty engaging in goal-directed cognition and behavior when distressed, (e) unwillingness to accept certain emotional responses, (f) lack of access to strategies for feeling better when distressed). Higher scores indicate more difficulties in emotion regulation. According to the preliminary findings, the DERS shows high internal consistency, good test-retest reliability, and adequate construct and predictive validity in undergraduate student samples [18].

Anger reaction is assessed using the Dimensions of Anger Reactions Scale-5 (DAR-5 [28];. This shortened version aims to measure five anger experiences (frequency, intensity, duration, antagonism towards others, social relation interference) of the past 4 weeks on a 5-point Likert scale ranging from 1 to 5. The DAR-5 has shown strong internal reliability and good validity in a college student sample with and without a history of trauma exposure [29] and in a male veterans sample with PTSD [28].

Additionally, substance use within the last 30 days will be assessed by using parts of the Addiction Severity Index Lite (ASI-Lite [21]). Participants will be asked in an interview to indicate how many days within the last 30 days they have used a given list of substances, the form of drug application, the age when they first started using the substance regularly, and for how many years they used the substance regularly [21]. The ASI-Lite is a short version of the ASI, which has shown to be a reliable and valid instrument with a wide range of clinical and research applications [30].

Anxiety is assessed using the General Anxiety Disorder (GAD-7 [31]) questionnaire. The GAD-7 is a brief seven-item self-report questionnaire, ranging from 0 to 3, that aims to identify probable cases of generalized anxiety disorder (GAD) and its severity in clinical practice and research by assessing the patients’ health status over the past 2 weeks. It has shown to have good internal consistency, test-retest reliability, and criterion, construct, and factorial and procedural validity in clinical settings [31].

The Patient Health Questionnaire-9 (PHQ-9 [32]) is the depression module of the full Patient Health Questionnaire (PHQ). The PHQ-9 aims to measure depression and its severity over the past 2 weeks in clinical practice and research. It consists of nine items plus an additional item if problems were checked off on the other items, and each of the 9 DSM-IV criteria are ranging from 0 to 3. Its measurement of depression severity has shown to be reliable and valid in clinical settings [32].

The Post Migration Living Difficulties (PMLD [33] checklist assesses the current post-migration stressors of asylum seekers. It is a self-report questionnaire with 24 items measuring the severity of different post-migration problems on a 5-point scale. It comprises difficulties experienced in the host country over the past 12 months or since arrival, if less than 12 months ago [33]. The items each measure a different experience, making reliability not relevant [34]. However, research has shown that the checklist can differentiate between asylum seekers and refugees with different residency statuses and can be used as a predictor of mental health in displaced population samples [33, 35, 36].

Quality of life is assessed using the WHO Quality of Life Scale Abbreviated Version (WHOQOL-BREF [37]). The WHOQOL-BREF is a 26-item self-report questionnaire including four domains of quality of life (physical health, psychological health, social relationships, environment), overall quality of life, and general health. It assesses the quality of life of the past 2 weeks on a 5-point Likert scale ranging from 1 to 5. The WHOQOL-BREF has shown good internal consistency, test-retest reliability, discriminant validity, and content validity in different subject samples with and without current health problems [37].

We will monitor and assess (serious) adverse events with a standardized report form during post-intervention and the 3-month follow-up. Any adverse events or reactions that are considered to be related to the intervention will be recorded, managed, and reported to the study coordinator. Serious adverse events will be reported to the ethics committee within 24 h of occurrence, and the study will be terminated prematurely.

The study assessors were trained to conduct the standardized assessment procedures prior to the start of the study.

#### Plans to promote participant retention and complete follow-up {18b}

To reduce dropout and missing data, reminders to participate in the intervention and monetary reimbursement of participants for the completion of each assessment (€35 per assessment) will be realized continuously.

#### Data management {19}

The data management will be performed at the Centre for Interdisciplinary Addiction Research (CIAR) at the University of Hamburg, Germany, which has successfully coordinated a substantial number of clinical studies as well as large multi-center trials. CIAR is responsible for the installation and monitoring of an overarching data handling concept and the integration of institutional policies of all project partners. An international advisory board will provide advice on methodology. Data integrity and plausibility will be assured by a combination of (i) tailored sets of suitable (and documented) algorithms and (ii) data rating by project associates following a protocol (including the rating of text entry). The Department of Medical Biometry and Epidemiology at the University Medical Centre Hamburg-Eppendorf will conduct the statistical analysis.

#### Confidentiality {27}

All study-related information will be stored securely at the study site. All participant information will be stored in locked file cupboards, and only the study personnel will have access to the cupboard. All reports and administrative forms will be identified by the 6-digit identification (ID) number to maintain participant confidentiality. All records that contain names or other personal identifiers, such as locator forms and informed consent forms, will be stored separately from the study records identified by code number. Personal data will not be transferred to the coordinating body except when necessary in the case of an SAE. All local databases will be secured with password protection. Documents that link participant ID numbers to other identifying information will be stored in a separate, locked file.

The study data will be collected anonymously via tablets. Anonymization will be implemented in accordance with the German Federal Data Protection Act. Each study participant will receive a 6-digit identification (ID) number. The anonymized data will be stored on a secure server of CIAR, which will perform research data management and data cleaning. Access to research data will only be granted to the staff involved in the cleaning and analysis of research data. Project partners will delete all personally identifiable data 5 years after the completion of the project.

#### Plans for collection, laboratory evaluation, and storage of biological specimens for genetic or molecular analysis in this trial/future use {33}

There will be no biological specimens collected.

## Statistical methods

### Statistical methods for primary and secondary outcomes {20a}

#### Primary data analysis

Data on all randomized study participants will be analyzed on an intention-to-treat basis. A mixed model for repeated measures (MMRM) will be applied, using the mean of all measured post-training scores of the outcome, adjusted for the pre-test score (alpha = 0.05, two-sided).

#### Safety

A *χ*^2^ test will be applied to compare the number of SAE between the two groups. Severity and relationship to treatment will be descriptively summarized in tables by group.

### Interim analyses {21b}

An interim analysis is not planned.

### Methods for additional analyses (e.g., subgroup analyses) {20b}

Subgroup analyses are not planned.

### Methods in analysis to handle protocol non-adherence and any statistical methods to handle missing data {20c}

The handling of missing values will be based on the full information maximum likelihood method (FIML) as ITT sensitivity analysis. Further, a per-protocol analysis will be performed on the subsample of participants with complete data and no protocol violation.

### Plans to give access to the full protocol, participant-level data, and statistical code {31c}

To ensure long-term access, data suitable for sharing and future reuse will be kept in citable form (with DOI) for at least 10 years. Data sharing is planned 2 years after the database closure (the final dataset will contain all predefined criteria, scores, and subscales) and after the publication of the primary and secondary outcomes in scientific journals. Open Access will follow the international standard formats, e.g., set out in the “Guidelines on Open Access to Scientific Publications and Research Data in Horizon 2020.”

### Oversight and monitoring

#### Composition of the coordinating center and trial steering committee {5d}

The Centre for Interdisciplinary Addiction Research (CIAR) at the University of Hamburg, Germany, will be responsible for the coordination of the trial. The CIAR prepares and coordinates the tablet-assisted assessments and coordinates the data management across all study sites. CIAR will also support the recruitment for all sites and initiate a continuous exchange of information between the project partners through regular online or in-person meetings at defined project stages and milestones (e.g., implementation, recruitment, and analysis). The PI is responsible for the submission of the formal ethical requests to the independent ethics committee and for the abidance of patient rights. Investigators at the different study sites are responsible for appropriate reporting of serious adverse events (SAE).

#### Composition of the data monitoring committee, its role, and reporting structure {21a}

Data safety is monitored by CIAR at the coordinating site. It will ensure adherence to the protocol and monitor the progress of the trial and provide the funding agency with information and advice, if requested.

#### Adverse event reporting and harms {22}

A serious adverse event (SAEs) for this study is any untoward medical occurrence that is believed by the investigators to be causally related to the study intervention and results in any of the following: life-threatening condition (that is, immediate risk of death), severe or permanent disability, and prolonged hospitalization. SAEs will be collected after the participant has provided consent and enrolled in the study. All SAEs occurring after the entry into the study will be recorded. To ensure participant safety, every SAE, regardless of suspected causality, occurring after the subject has provided informed consent until the subject has stopped study participation, must be reported by the sites to the PI within 24 h of learning of its occurrence. The investigators of the sites take the responsibility for appropriate reporting of SAEs. In case of a SAE, discontinuation will be decided by consensus of PI, local investigator, and the Study Safety Board. The main concern will be the safety of the study subjects.

#### Frequency and plans for auditing trial conduct {23}

No external audits are planned for the study. The Project Management Group will meet weekly to review the trial conduct throughout the data assessment period. The Scientific Adivsory Board and the Refugee Advisory Board will meet at least yearly to review the trial conduct.

#### Plans for communicating important protocol amendments to relevant parties (e.g., trial participants, ethical committees) {25}

Any modifications to the protocol which may impact the conduct of the study and the potential benefit of the patient or may affect patient safety, including changes in study objectives, study design, patient population, sample sizes, study procedures, or significant administrative aspects will require a formal amendment to the protocol. In the case of such amendments, they will be communicated to all relevant parties including trial registries. Study information documents provided to the participants will be updated according to the protocol modifications to inform participants about the changes.

#### Dissemination plans {31a}

The results of the trial will be presented at international conferences and will be published in an open-access journal.

## Discussion

This study will be the first RCT on a culturally sensitive transdiagnostic intervention for trauma-exposed refugees with hazardous substances or SUD. As there is no gold standard for the treatment of the target group, the intervention (STARC-SUD) will be compared to treatment as usual.

This trial has limitations that should be considered in evaluating the results. All data will be collected using self-report measures. This can introduce measurement errors and information bias. While the outcome adjudicators will be blinded for the assignment of interventions, assessors will only be blinded for the baseline assessment for reasons of feasibility. Unblinded assessors may introduce measurement bias in the measurement of outcomes. Another limitation of this trial is that we only include male participants. Hence, the results might not be generalized to the population of female participants. Study participants will be restricted to those who speak a limited number of languages, e.g., Arabic, Farsi, Dari, English, or German, which will further limit the generalizability of the results. Finally, there is no long-term follow-up, so we will not be able to determine long-term efficacy. Nevertheless, it can be assumed that the trial will yield important insights into the feasibility and efficacy of interventions for a particularly vulnerable group of patients.

## Trial status

Recruitment for the RCT commenced on September 24, 2020, and will continue for at least 36 months.

## Data Availability

Anonymized data can be obtained from the first author upon reasonable request.
